# Supplementary material for: Discovery of tumoricidal DNA oligonucleotides by response-directed in vitro evolution
Source: Commun Biol. 2020 Jan 15;3:29. doi: 10.1038/s42003-020-0756-0 (PMC6962221; doi:10.1038/s42003-020-0756-0)
Supplement: Supplementary file 1 — Supplementary Information [file 42003_2020_756_MOESM1_ESM.pdf]

Supplementary Figures

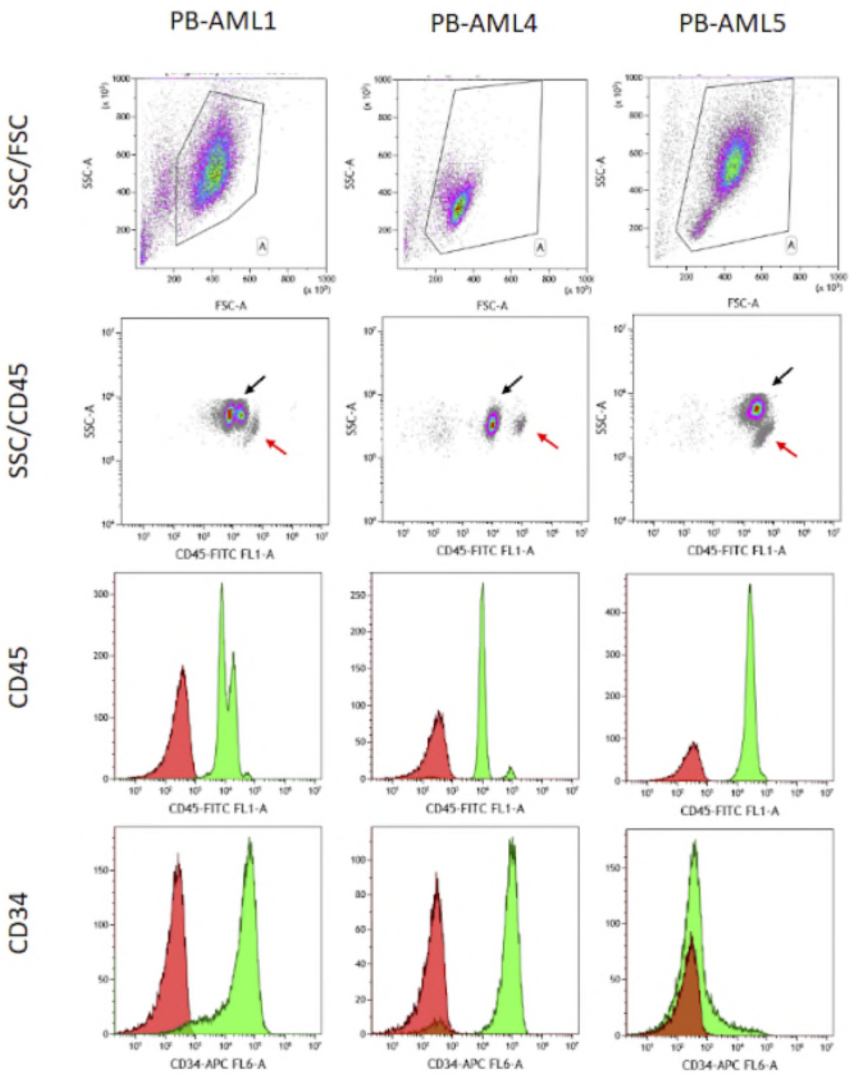

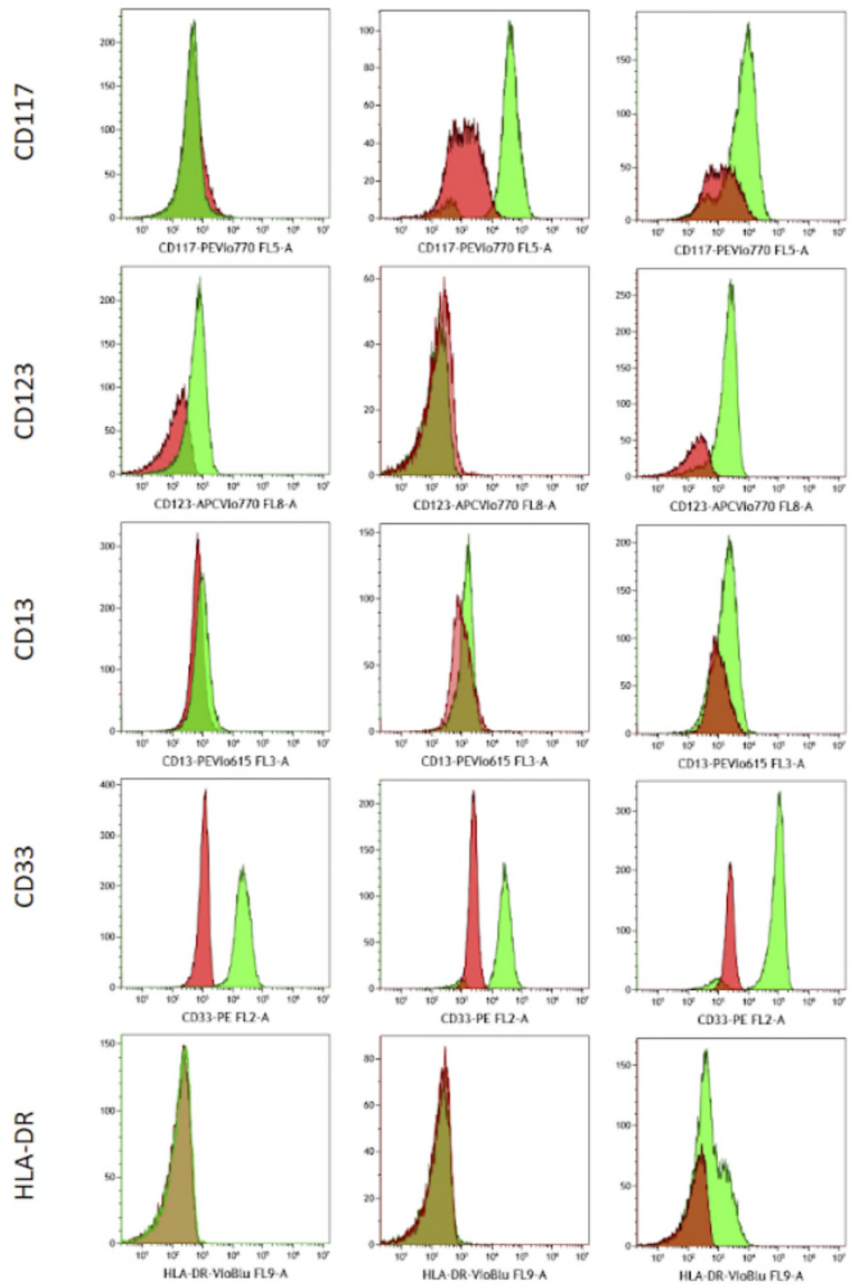

Supplementary Figure 1. Immunophenotyping of cells from AML patients. Ficoll-isolated primary AML cells were stained with blasts markers CD117, CD34, HLA-DR and CD123 (green histogram. isotype staining appear in red). CD33 and CD13 (green histogram isotype staining appear in red) were used as myeloid markers. In the CD45/SSC scatter the blast population is marked by a black arrow and the normal cell population is marked by a red arrow.

PB-AML4

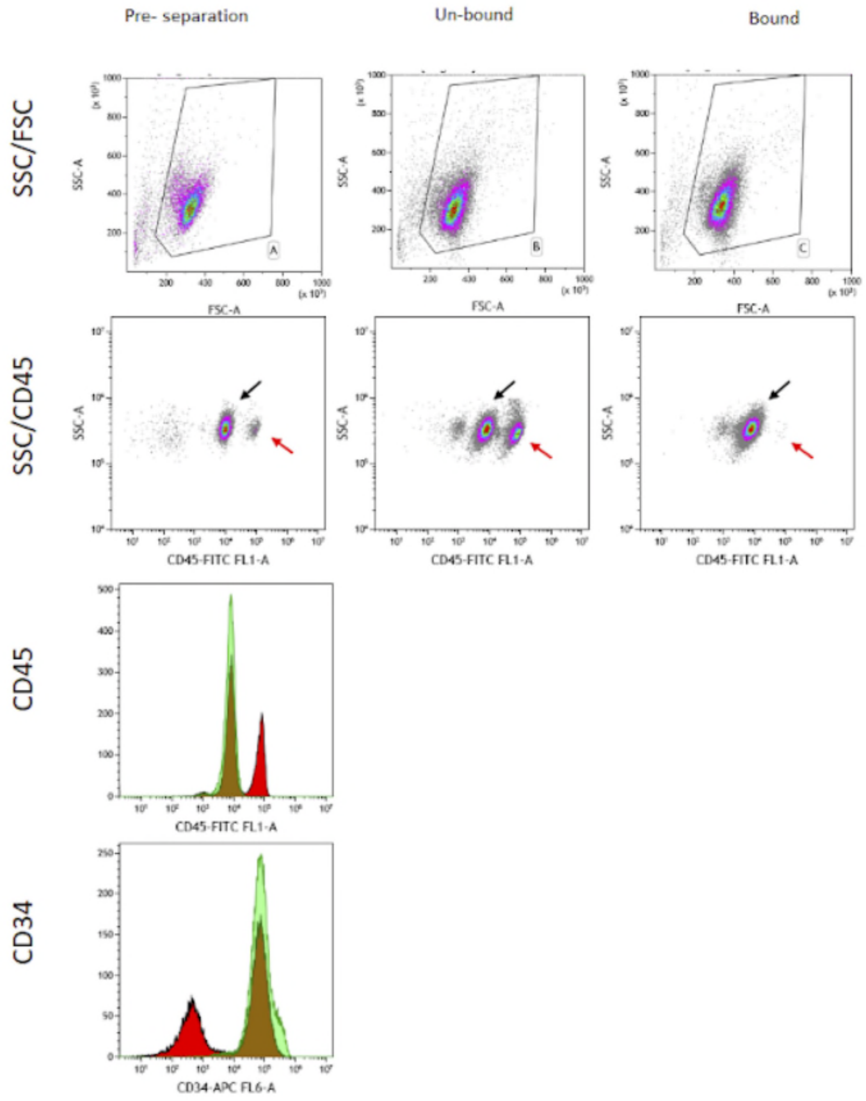

Pre- separation
Un-bound
Bound
SSC/FSC
SSC/CD45
CD45
CD34
SSC-A
FSC-A
CD45-FITC FL1-A
CD34-APC FL6-A

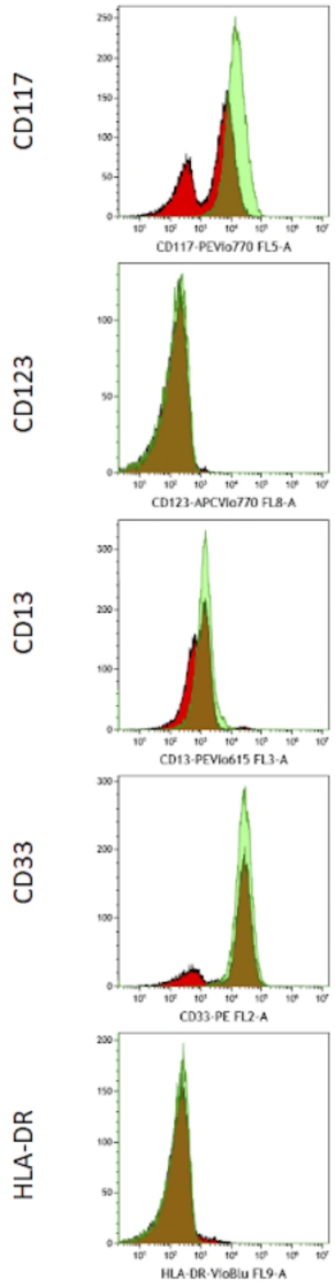

Supplementary Figure 2. Blood before and after sorting. Example of AML sample (PB-AML4) characterized by immunophenotyping as CD117 bright were magnetically separated via anti CD117 magnetic beads' column. The CD117 positive population (bound) was compared via CD45/SSC to the CD117 negative (unbound) population.

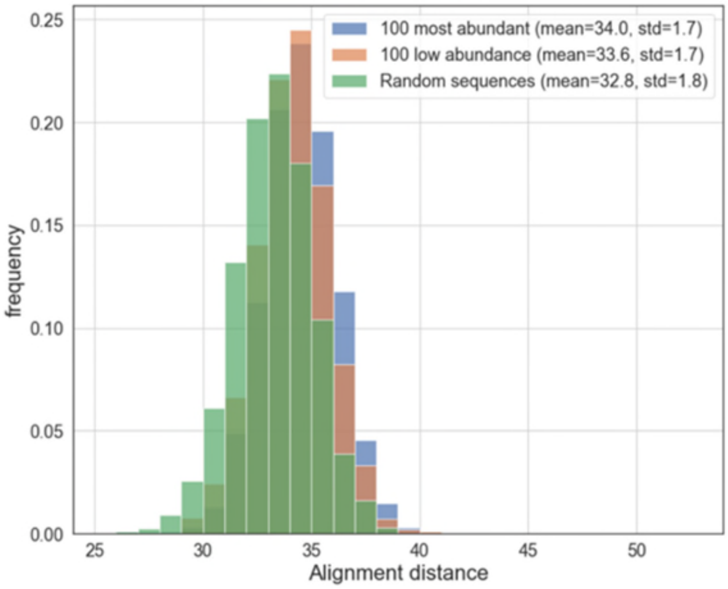

Supplementary Figure 3. Sequence homology analysis.

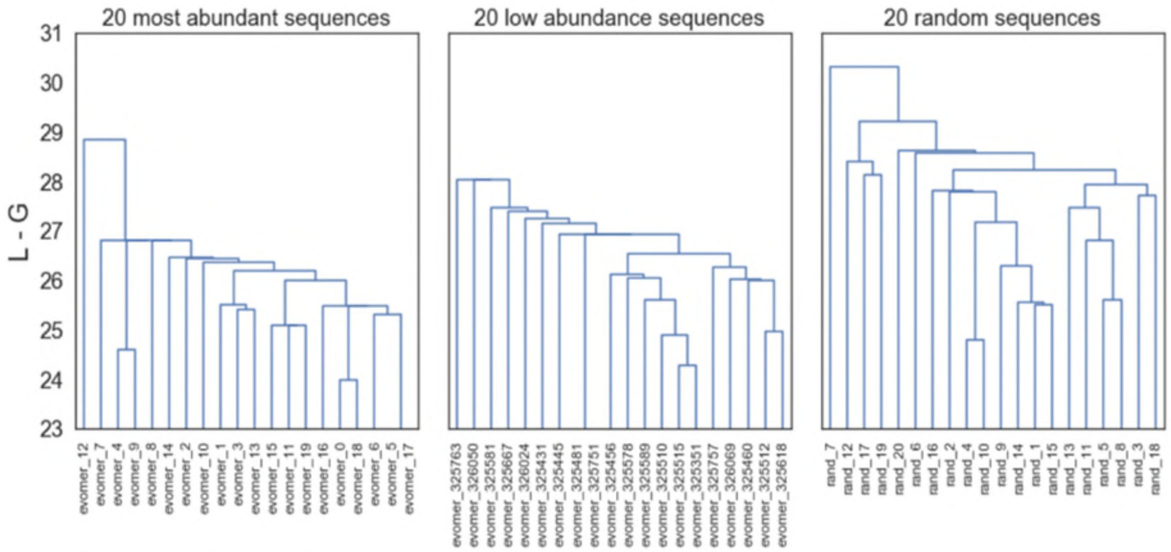

Supplementary Figure 4. Dendrogram analysis of sequence homology.

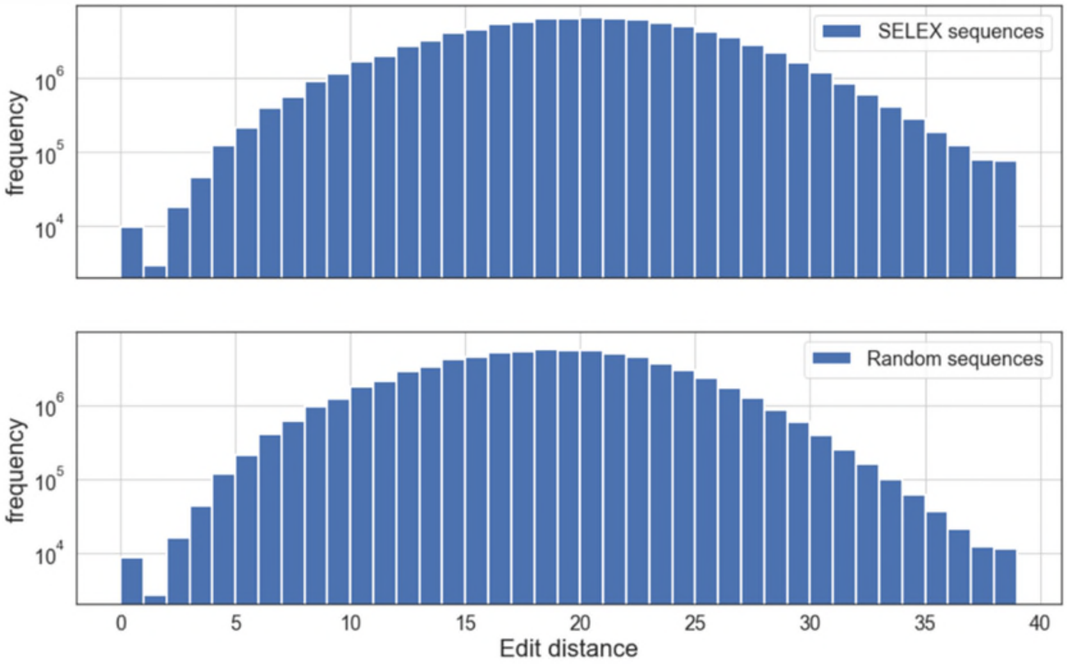

Supplementary Figure 5. Secondary structure homology analysis.

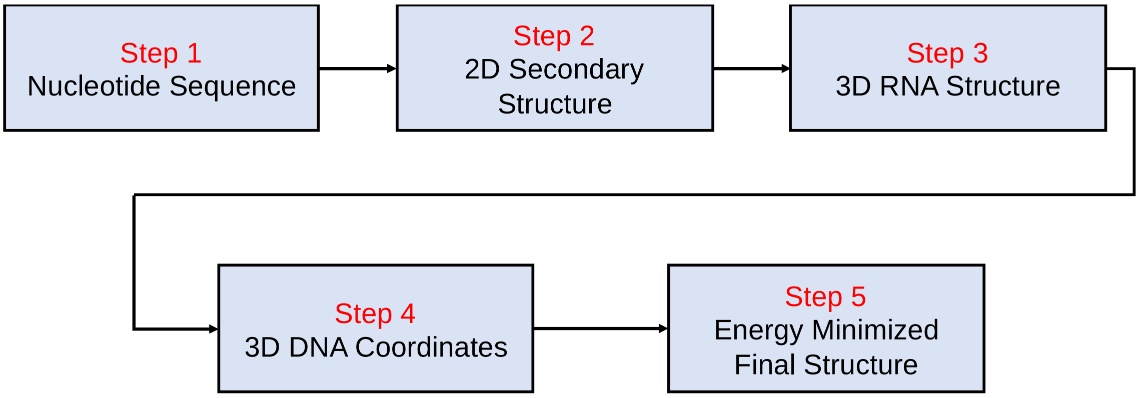

Supplementary Figure 6. Workflow for ssDNA structure simulation and prediction

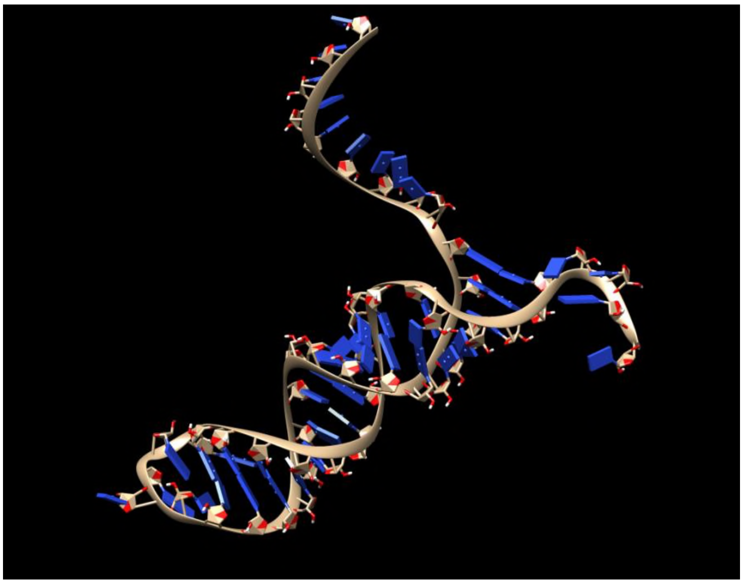

Supplementary Figure 7. Predicted 3D RNA structure

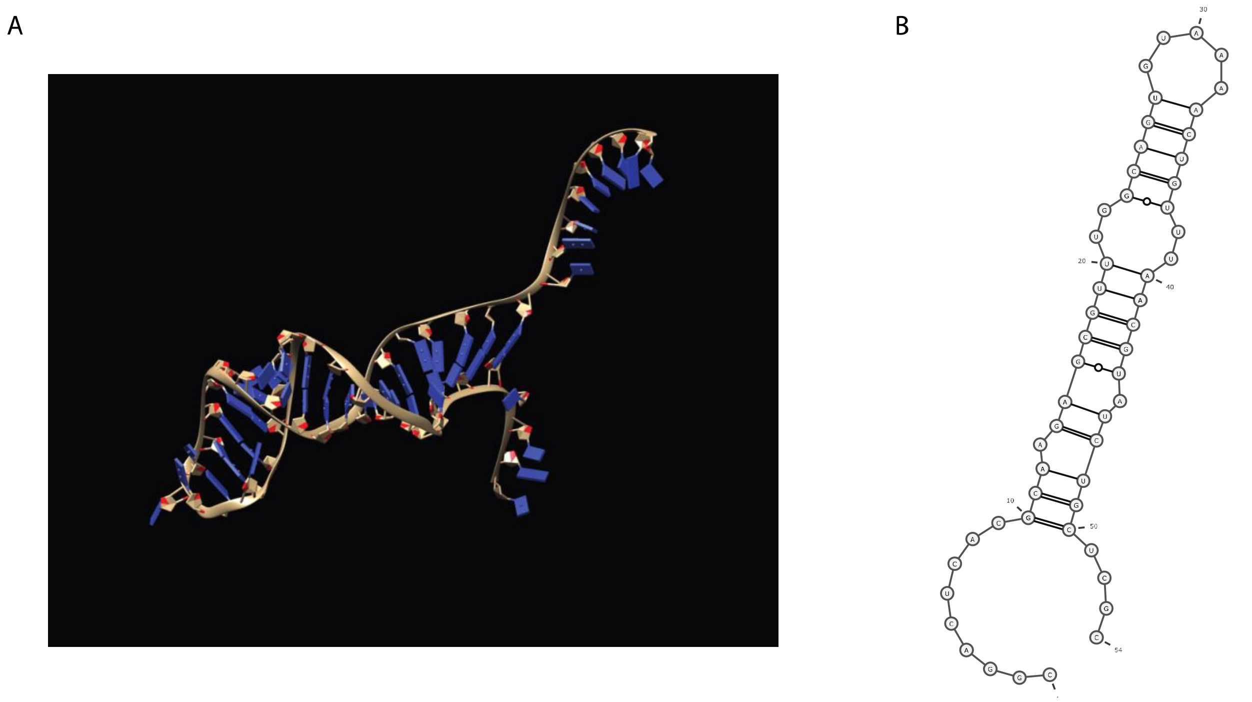

Supplementary Figure 8. A, Structure of ssDNA converted from the RNA model. **B,** 2D illustration showing base pairs in the predicted ssDNA structure

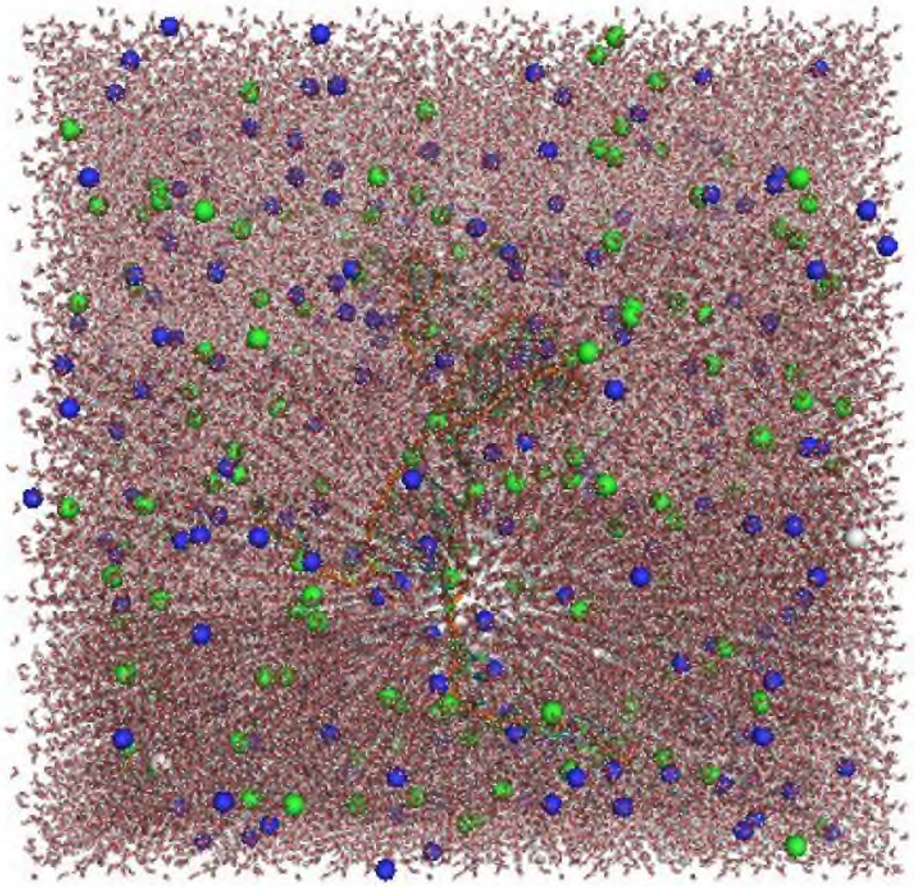

Supplementary Figure 9. ssDNA in the water box with various ions.

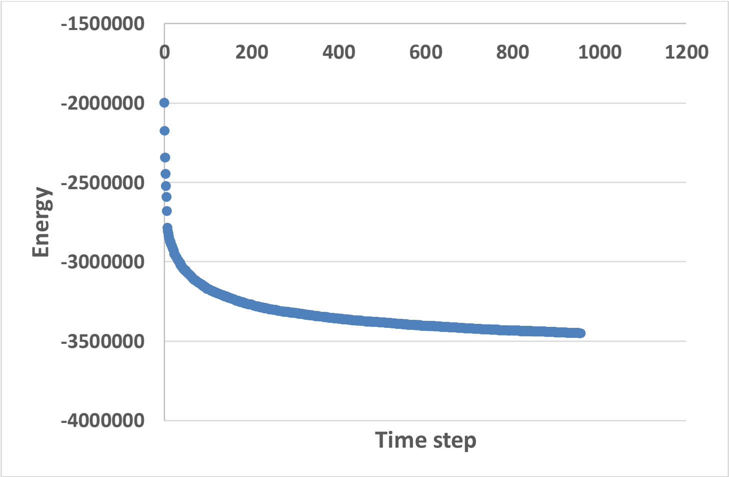

Supplementary Figure 10. Energy change during simulation. The energy of the simulation system has been minimized and reached equilibrium.

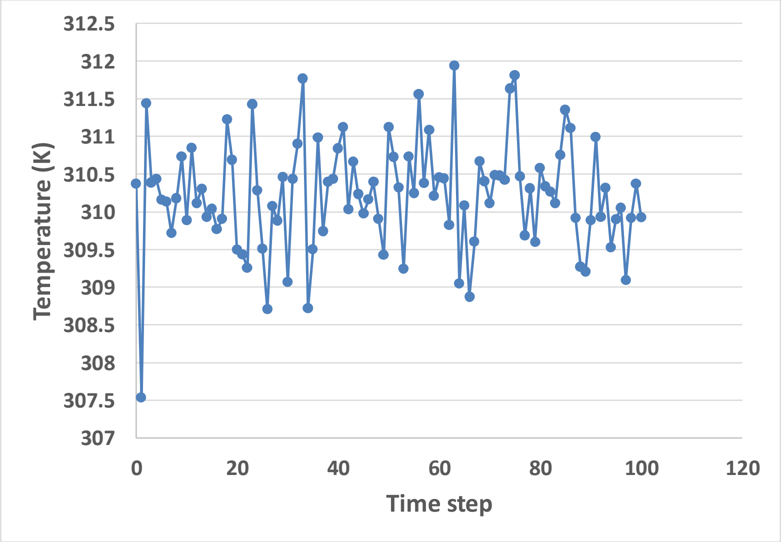

Supplementary Figure 11. Temperature change during simulation. The temperature has reached equilibrium and is kept at around 310.15 K.

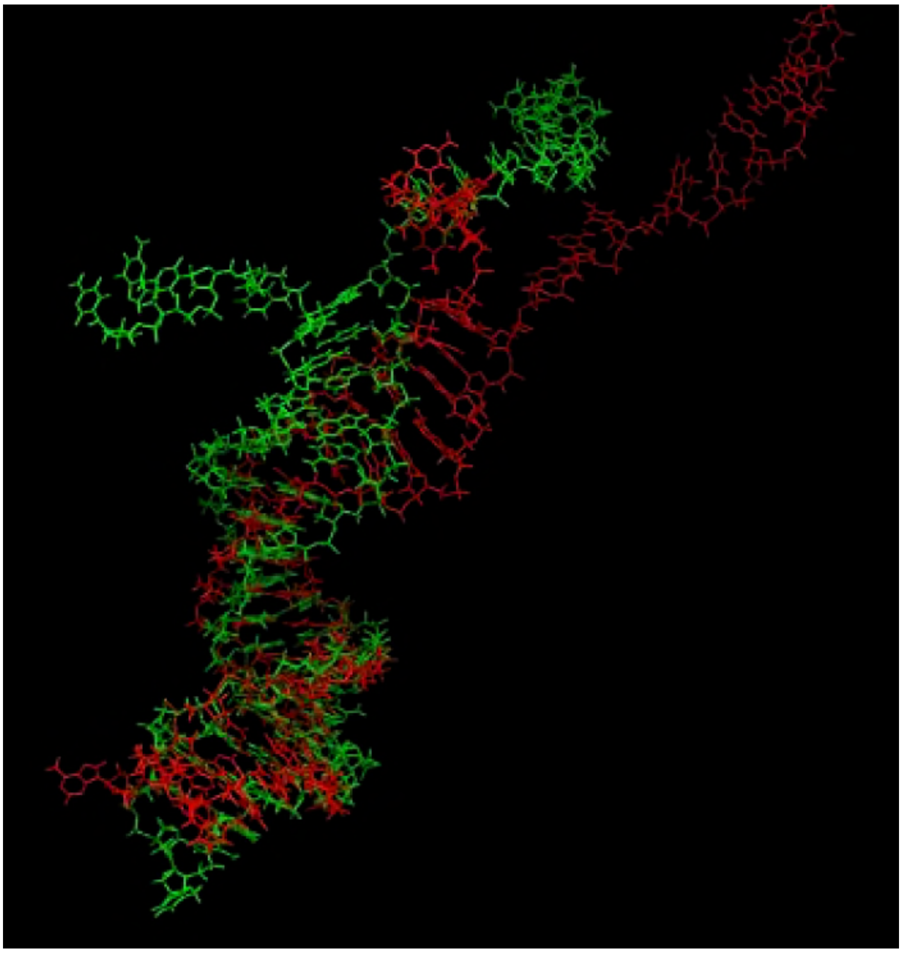

Supplementary Figure 12. Comparison of ssDNA structures before (green) and after (red) energy minimization.

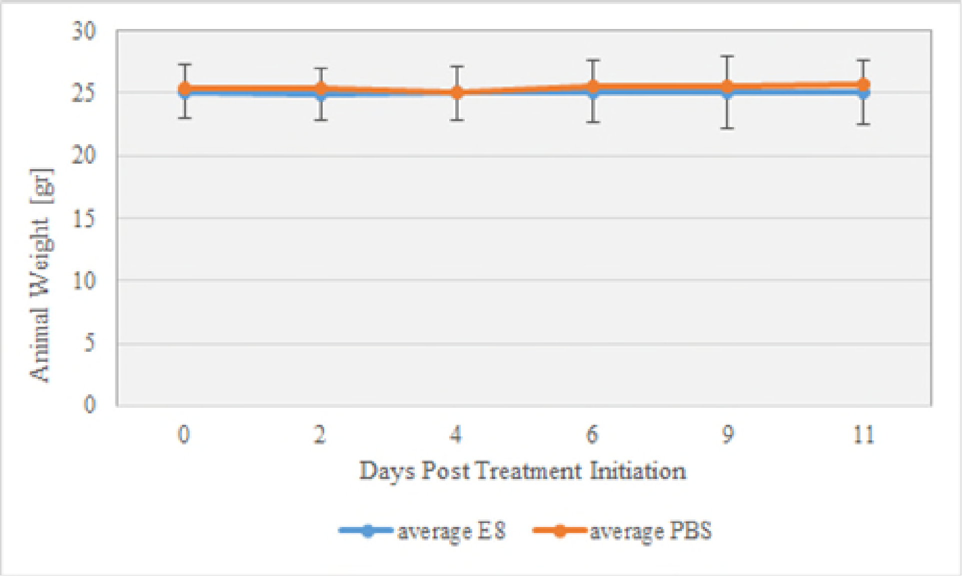

Supplementary Figure 13. Mice weight during the course of treatment

Animals were measured prior to each injection and two days after the final injection. Weight is represented in grams.

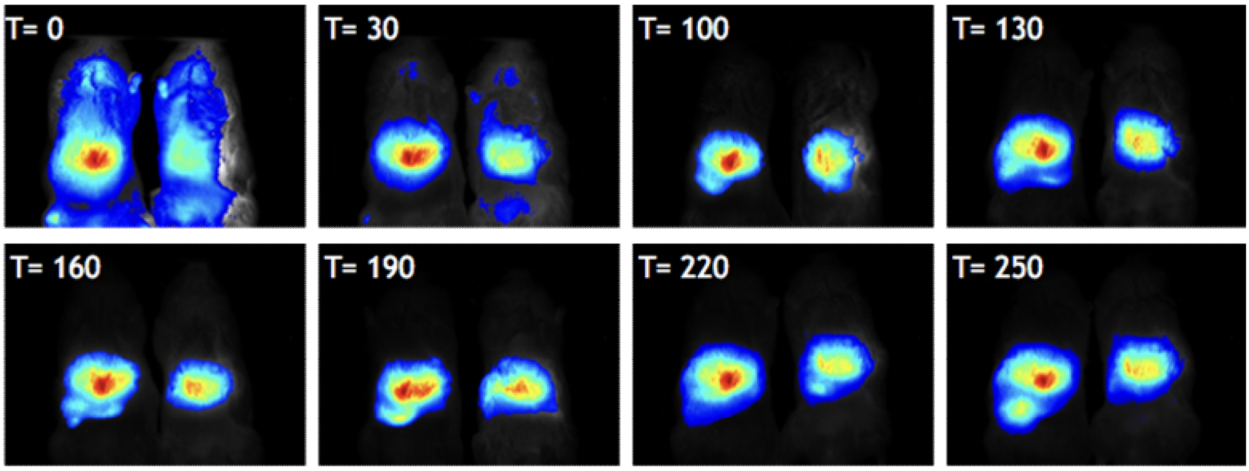

Supplementary Figure 14. Distribution of control oligo in tumor-bearing mice. After as early as t = 30 min, signal from the oligo is restricted mostly to the liver; at t = 100 min it also appears from the kidneys. Oligos in this experiment were not PEGylated.

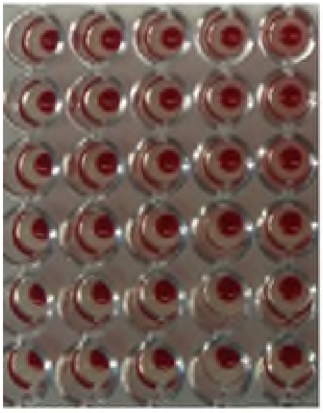

vehicle	50uM E8	1uM E8	0.1uM E8
vehicle	50uM E8	1uM E8	0.1uM E8
vehicle	50uM E8	1uM E8	0.1uM E8
vehicle	10uM E8	0.5uM E8	0.01uM E8
vehicle	10uM E8	0.5uM E8	0.01uM E8
vehicle	10uM E8	0.5uM E8	0.01uM E8

Supplementary Figure 15. E8 Blood agglutination testing

Blood from a healthy donor was incubated with 0.01, 0.1, 0.5, 1, 10 and 50uM of three top leads. The table on the left depicts the test in the wells in the photo to the right. No agglutination was observed for any lead. Samples on rightmost column are untreated.

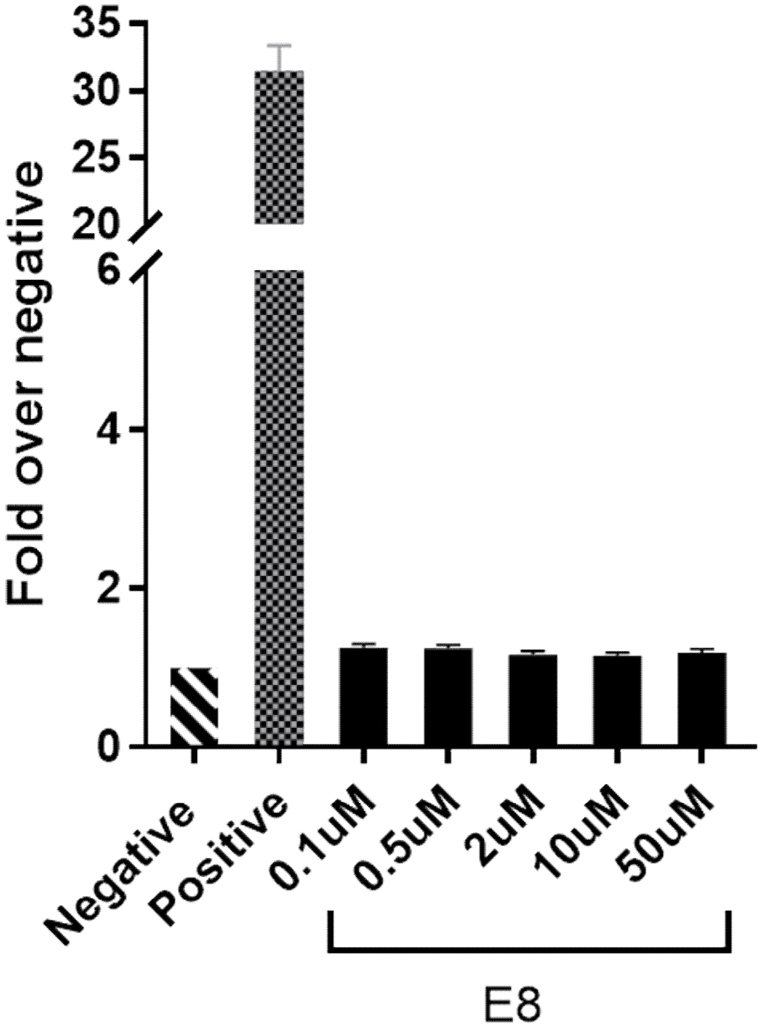

Supplementary Figure 16. E8 Red blood cell hemolysis.

Red blood cells of the healthy donor were incubated with 0.1, 0.5, 2, 10, 50 uM of top three leads, including E8. Hemolysis was measured as absorbance at 540 nm. Ultrapure water was used as positive control. No hemolysis was observed for top leads.

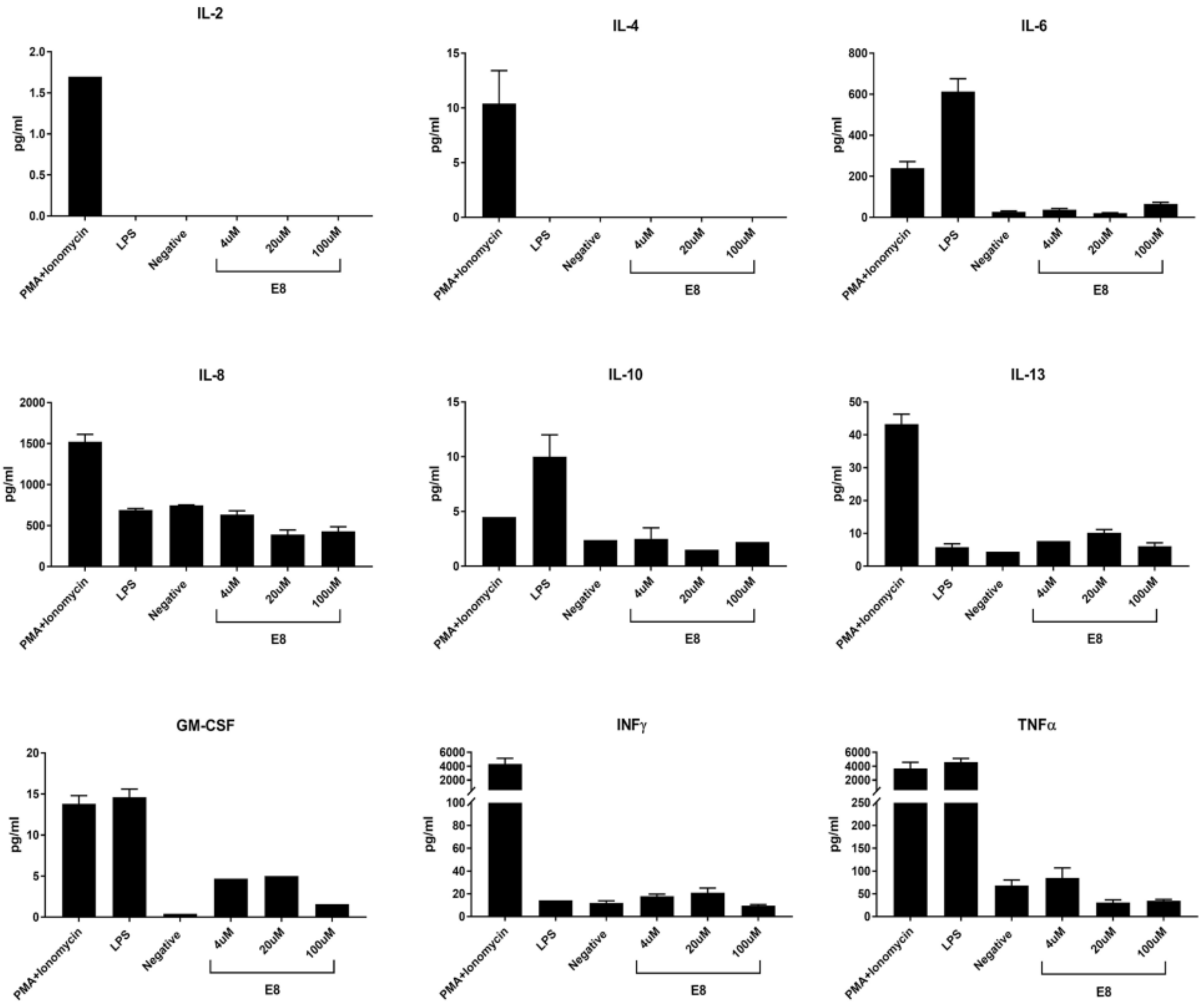

Supplementary Figure 17. Cytokine antibody array for detection of twelve cytokines release after incubation of top leads with PBMCs of a healthy donor.

PBMCs of a healthy donor were incubated for 3 and 6 hours with 4, 20 and 100 uM of three top leads, including E8. As positive control lipopolysaccharide and a combination of PMA and ionomycin were administered to the cells.

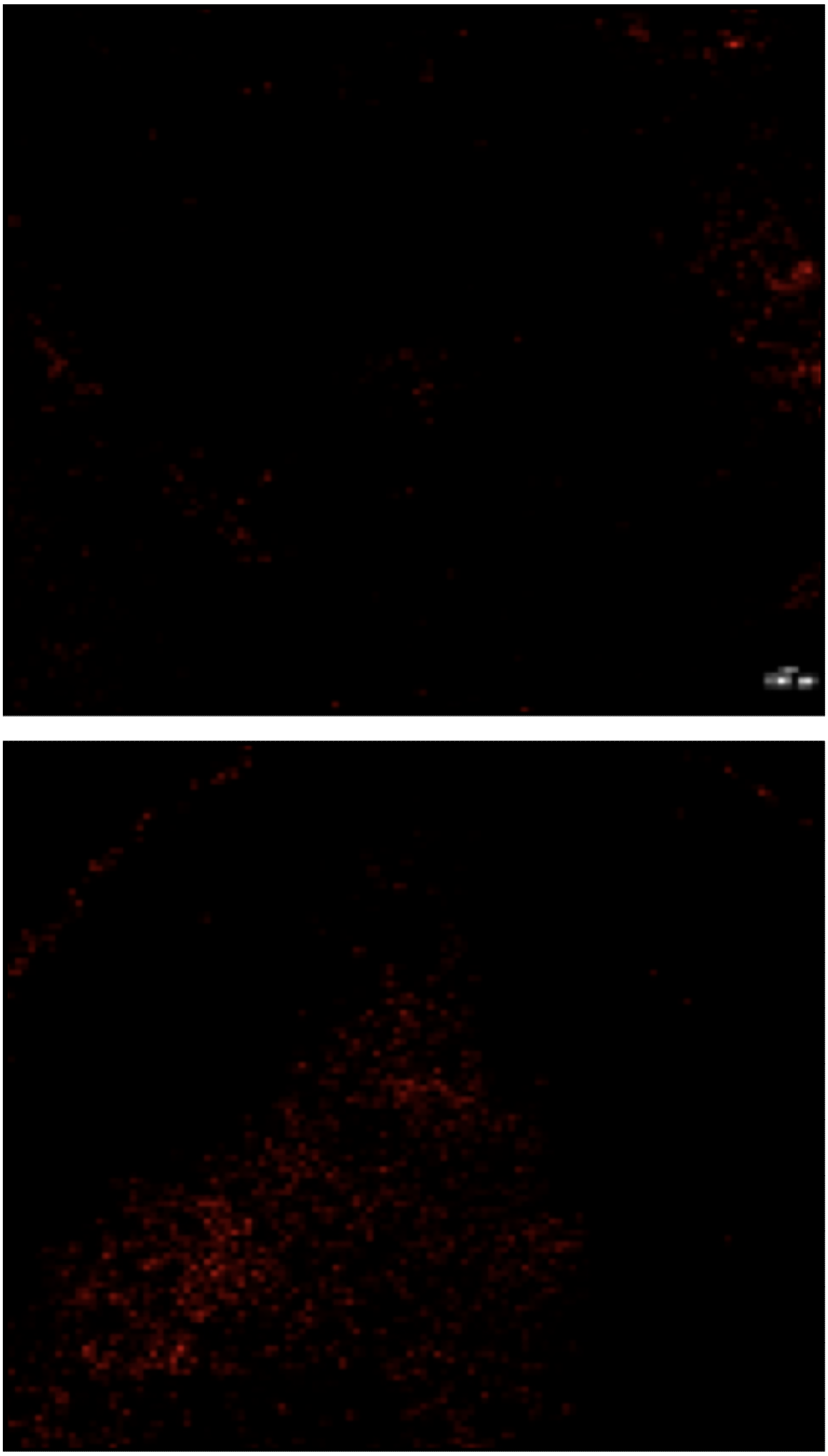

Supplementary Figure 18. Magnified TUNEL results (originally Fig. 3H,I).

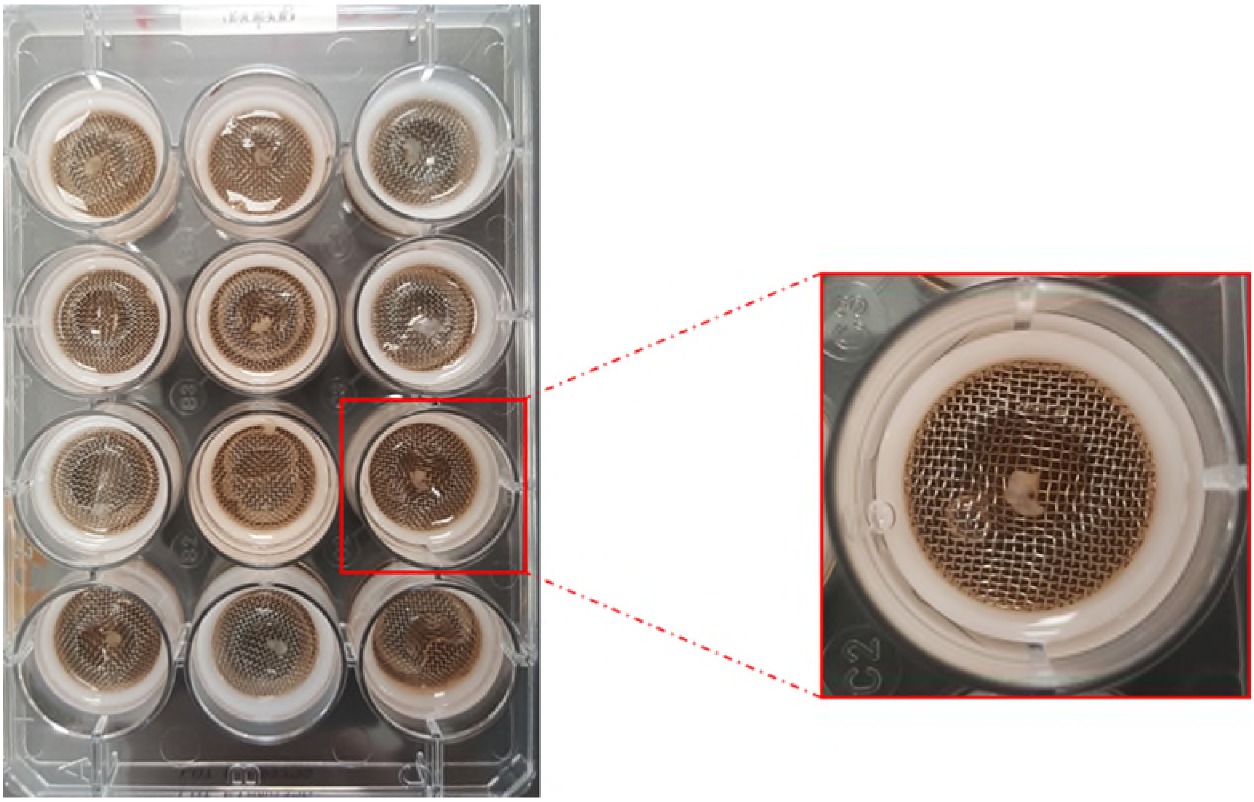

Supplementary Figure 19. Ex vivo organ culture (EVOC) system example for primary solid tumor efficacy studies.

A 24 well plate of 250um wide tissue slices, each maintained on a special scaffold in culture medium at high oxygen conditions. Cancer cells are supported by physiologically relevant microenvironment of the primary tumor and are kept viable for up to 14 days, pending tissue source. Following efficacy study H&E staining are performed for each tissue slice in each well

Supplementary table 1: Screened sequences

Oligo ID	Oligo sequence (5’-3’)
E1	TAAGGGTAGCAATGCGTTAGTCGCTTAAAATTCGATTTGCGCATAACACCTCAT
E2	CACAAGGGCAGTACTCTCGAGATTAATGTGTACATGCACTCGCGAAATGTTGAG
E3	TGCGTAGTATAACCGCTAATCAATCGTACAATGTAACCTTGACCGCACACGGCC
E4	CACACAGCGACAGCATAGTCTCGTACTGGCTTAAAACATGAAGTTGCGATTAAT
E5	AACACCGCTATCTATCGTCATGTCAGGCGTGTACTTGACTTACATCTATTGACC
E6	ACATCACATTTGCCTGCGATCAAGCTAACACGCATGATACCATCATGATTAACC
E7	TTGCTGCTCGGATCAGGCAAGACGCTACCCACAACTCGGTTTGTAAGACTACAC
E8	CGGACTCACGCAAGAGCGTTTGGCAGTGTAAAACTGTTTAACGTATCTGCTCGC
E9	ATTGCGAGATCACTATGTTTTAGTCTAGGCTAGCACGCTACTTGGGACTGTAGA
E10	CACGACGAGATACCGTGGTCCTTTGGACGCGAATGTCATTTAGCACTTAGCATT

Supplementary notes

Supplementary note 1: Cell-SELEX and phase exit determination

Supplementary note 2: Clustered library synthesis and QC

Supplementary note 3: Immunophenotyping

Supplementary note 4: Sequence structural homology analysis

Supplementary note 5: Simulation and prediction of 3D structure

Supplementary note 6: Animal safety data

Supplementary note 7: E8 in human blood

Supplementary note 8: TUNEL results (magnified images)

Supplementary note 9: EVOC experimental design

Supplementary note 10: Functional reporter selection

Supplementary note 1: Cell-SELEX and phase exit determination

Cell-SELEX was performed as follows. An ssDNA library constructed of a random core flanked by constant regions is folded in the presence of constant region-complementary oligonucleotides (termed caps). Folding was carried out by incubation at 95 □ for 5 min, cooling on ice for 10 min, and an additional 10 min incubation at 37 □. Folded library and cells were incubated together in the target cell medium supplemented with 10% human serum for 1h. Library concentration in the incubation step was set to 500 nM. After each round, the sample was washed to dilute unbound candidates 10^4- fold for the first selection round and 10^6-fold from the second round forth. To prepare the next round’s input library, the bound fraction was eluted by incubation at 95 □ for 10 min. From the 2nd round on a negative selection was added. The eluted library was folded again and incubated with the non-target cells as described above, this time the unbound fraction is taken as an input for an **asymmetric PCR (aPCR)** process. ssDNA was purified from the aPCR product using preparative HPLC on an Agilent 1100 instrument. Samples of output libraries from all rounds were stored for evaluation.

To evaluate the success of the overall cell-SELEX process, output libraries from all rounds of the cell-SELEX were amplified by aPCR using fluorescently-labeled forward primers, and a binding assay on the target cells was performed comparing all output libraries with the initial random library as a negative control. Flow cytometry was performed on two instruments, Becton-Dickinson Accuri C6 Plus flow cytometer equipped with 488 nm and 630 nm lasers, and a Beckman-Coulter Cytoflex flow cytometer with a B5-R3-V5 laser configuration.

The functional selection stage as presented in this work adds a constraint that forms a selective pressure in the process. That is, for the successful “survival” of an oligo candidate and identification, it must stay attached to the apoptotic cell through the incubation time and while sorting. It means, that it is not enough for the candidate to have a functional quality, it should be a strong enough binder. Unfortunately, we are limited in the number of candidates we can screen, restricted by the number of beads available to us. Since the initial library holds 5-6 orders of magnitude more candidates than we can screen at the functional stage, it is important to narrow down the list of candidates to increase the chances of the process to succeed. To this end, we use the selex process to shed candidates that would not be able to survive the functional process. Nevertheless, it is important not to commit to many rounds of selex. While driving the population into binding we are losing many candidates that while they are not the strongest binders found in the library, they could survive the functional process and are legitimate functional candidates. On the other hand, in order to drive the library towards more specificity, it is important to incorporate a few negative selections within the rounds. To this end and as a compromise between the conflicting interests, we decided to get into the functional assay using the input of the 3rd round of the cellSelex process.

Supplementary note 2: Clustered library synthesis and QC

Upon going into the functional stage of the selection scheme, the input binding round of choice is loaded on beads in a fashion such that each bead will be covered with many copies of the oligo candidate. This is done in order to amplify the signal the oligo generates by creating an effective local high concentration of the candidate. A bead can hold several candidates. Obviously, the more candidates every bead hold a bigger variety of candidates can be screened for a given number of beads. The tradeoff would be the effective concentration for each of the candidates. To generate the clustered beads we used the Ion Proton sample prep Ion PI™ Hi- Q ™ OT2 200 Kit and an Ion OneTouch™ automated sample prep system. The protocol supplied with the kit is optimized for the proton sequencing technology, where the creation of a high percentage of monoclonal beads is a priority. That is a recommendation of 6-8 ul of a 100 pM stock should generate about 10% templated beads. We chose to go for the increased variety, using 1ul out of 2 nM stock and got 40% templated beads. The Poisson distribution that fits this percent of templated beads shows that > 0.75 of the population of templated beads are monoclonal, ~ 0.195 of the templated beads are biclonal and only ~ 0.05 of this population holds 3 oligo candidates or above. With the number of beads per reaction as the limiting factor, this translates to ~ 642 $*10^6$ oligos represented over ~ $500*10^6$ beads. Of course, unless one goes into the OneTouch process without amplifying the initial library the number of unique sequences represented on the beads would be smaller as there can be more than one copy of each of the candidates and therefore occupying more than one bead. With the exception of higher than the recommended amount of template, the Ion PI™ Hi- Q ™ OT2 200 Kit user manual instructions were followed. Enrichment QC was done using the Ion Sphere™ Quality Control Kit according to the manufacturer instructions. Ion spheres were labeled using Cy5 conjugated caps in order to help with their detection in the melody FACS.

Supplementary note 3: Immunophenotyping

Blood was freshly obtained from AML patients and run on ficoll gradient to isolate leukocyte fractions. Viable cells were counted using trypan blue. Leukocytes were divided into aliquots supplemented with FBS and 10% DMSO and were kept in liquid nitrogen. Immune phenotyping was done using antibody markers based on the EuroFlow suggested panels[1]. CD33 and CD13 markers were used to indicate myeloid cells, and blasts were determined by the expression levels of CD117, CD123, CD34, and CD45 **(Supp. Fig. 1)**. Wherever AML samples contained <95% blasts, a commercial kit (Miltenyi Biotec) for magnetic sorting with antibodies against the most predominant marker to enrich the blasts population **(Supp. Fig. 2)**. The kit was used precisely according to the manufacturer instructions.

[1] van Dongen et al., "EuroFlow Antibody Panels for Standardized N-Dimensional Flow Cytometric Immunophenotyping of Normal, Reactive and Malignant Leukocytes."

Supplementary note 4: sequence structural homology analysis

Sequence clustering & homology

The NGS reads for each sample were counted and sorted by abundance, followed by sequence clustering using VSEARCH Rognes et al, 2016) by a sequence similarity of 90% or higher and its abundance summed. Virtually all the sequences in each cluster were found to be close variants of the seed sequence, which generally comprises over 90% of the reads in the cluster. We interpret the variants as originating from PCR mutations or sequencing errors.

We could not find a meaningful homology between the most abundant sequences in the latest SELEX round. We used pairwise global alignment score as a similarity metric representing homology (pairwise2.globalxx in Biopython, see Cock et al, 2009). Below we plot the distribution of the pairwise distances between sequences in the 100 most abundant sequences, compared with 100 low abundance sequences and with random sequences. The most abundant sequences show a very small difference compared with the low abundance group (~0.25 standard deviations), and a slightly larger one with the random sequences (~0.7 standard deviations).

The most similar sequence to Evomer 8, for example, is Evomer 66. The alignment between them is shown below:

```
--CGGA--CTCAC-GC----AAGAGCGTT-TGGCA--GT-GTAA-AACTGT-TTAACGTATCTGCTCGC
  |  |  ||||| ||    ||||||||| | |||  |  ||   || ||  || ||||| ||  |
TAC--ATACTCACTGCTGTGAAGAGCGTTAT-GCAACG-AGT--GAA-TG-GTT-ACGTA-CT--T---
  Score=39
```

The two sequences share a 9mer and two 5mers. It is not higher than expected from random sequences in a statistically significant manner, and the sequences are also far from obviously homologous. This trend can also be seen in the following dendrogram analysis of sequences from 3 different pools: 20 most abundant, 20 least abundant, and 20 random oligos.

Secondary structure similarity

We investigated whether there are significant motifs in the secondary structure level. We defined the secondary structure distance in this context as the edit distance between the dot-bracket representations of two sequences (for example "…(((…..)))..”), after stripping the unpaired 5’ and 3’ ends. We calculated the secondary structure for all the 471917 sequences in the last SELEX round using ViennaRNA (Lorenz et al, 2011), comprising a total of 9097 unique stripped structures. We compared the distribution of pairwise distances between the unique structures for the SELEX sequences and for random sequences, and found no difference between the two as shown below. Attempting to cluster the structure together yielded no promising results.

Supplementary note 5: Simulation and prediction of 3D structure

The workflow for three-dimensional structure generation from sequence consists of five main steps:

1. ssDNA sequence
2. building the ssDNA secondary structure from sequence using Mfold
3. constructing refined equivalent 3D RNA models using Assemble2
4. translating the 3D RNA models into DNA models using Chimera
5. refining the final 3D ssDNA structure through minimization using GROMACS.

5.1 Secondary structure prediction for ssDNA

The first step is the prediction of secondary structures from DNA. The input consists of the DNA sequence:

5'-CGGACTCACGCAAGAGCGTTTGGCAGTGTAAAACTGTTTAACGTATCTGCTCGC-3'.

Starting with the nucleotide sequence, the secondary structures of the ssDNA molecules were predicted using Mfold, in which all possible secondary structures are approximated based on Watson-Crick base pairing and the most thermodynamically stable structure is selected. The initial sequence was selected as linear at a temperature of 37°C and ionic concentration of 150 mM Na^+ and 5 mM Mg^{2+}, computing only fold configurations within 5% from the minimum free energy, and considering a maximum number of 50 folds with no limit to the maximum distance between paired bases. In addition to the predicted secondary structure of the ssDNA this step provides the minimum free energy of $\Delta G = -16.30$ kcal/mol.

Files generated in this step:

File name	Description
**Secondary_structure_prediction.ct**	A .ct file describing secondary structure
**Secondary_structure_prediction.log**	A .log file containing information of the simulation

5.2 Three-dimensional structure prediction for ssRNA

In this step, the predicted secondary structure was used as a starting point to generate the 3D structures of the equivalent ssRNA models using Assemble2. The 3D structure was modeled and visualized using Assemble following a manual process of individually selecting the 2D helical and non- helical elements of the ssDNA molecule and translating the residues into equivalent 3D RNA models. The 3D RNA model was then refined using 100 iterations to remove geometric deficiencies and optimize the structural parameters such as bond length and angles, planarity of certain groups, non- bonded contacts, and restricted torsion angles. The refinement was achieved by the geometrical least squares method using the Konnert–Hendrickson algorithm as implemented in the Assemble2 program.

Files generated in this step:

File name	Description
**3D_ssRNA.pdb**	A .pdb file describing 3D structure of the RNA
**3D_ssRNA.log**	A .log file containing information of the simulation

5.3 Generation of 3D ssDNA coordinates

In this step, the refined ssRNA 3D structures were imported into Chimera, where hydrogen atoms were added and the ssRNAs modified into ssDNA 3D structures by: (i) identifying each uracil residue and replacing the H5 atom with a methyl group, and (ii) replacing the ribose sugar backbone with deoxyribose and manually renaming the modified uracil residues to thymine in the pdb file.

Files generated in this step:

File name	Description
**3D_ssDNA.pdb**	A .pdb file describing 3D structure of the ssDNA
**2D_ssDNA.pdf**	A .pdf file showing an image of base pairs in the ssDNA
**2D_DNA_dotbracket.txt**	A .txt file with dotbracket format describing base-pairing information

5.4 Energy minimization

The last step of our approach consists of further refinement of the ssDNA 3D structures obtained in 3.3. The output of this step consists of the final coordinate (.pdb) file in addition to all other intermediate files. Energy minimization was performed by steepest descent approach using GROMACS with Charmm27 force field. The ssDNA was placed in a solvent box consisting of water molecules, and Na+, Mg2+, Cl- ions at specified concentrations. The temperature was set at 310.15 K.

Files generated in this step:

File name	Description
**Final_ssDNA.pdb**	A .pdb file describing the energy minimized structure of ssDNA
**ssDNA_in_water.pdb**	A .pdb file describing the whole simulation system during energy minimization
**Energy_and_temperature.xlsx**	A .xlsx file with energy change and temperature change data during simulation
**energy_minimization.***	Intermediate files generated during simulation

5.5. Post-simulation analysis

Analysis of the final structural model reveals the following intra-molecular interactions.

Pair ID	Pair	ResID1	Base1	Base2	ResID2	Hbond #	W-C type
1	G-C	10	G	C	50	3	Y
2	C-G	11	C	G	49	3	Y
3	A-T	13	A	T	48	2	Y
4	G-C	14	G	C	47	3	Y
5	C-G	17	C	G	43	3	Y
6	G-C	18	G	C	42	3	Y
7	T-A	19	T	A	41	2	Y
8	T-A	20	T	A	40	2	Y
9	T-T	21	T	T	39	2	N
10	G-T	22	G	T	38	1	N
11	G-T	23	G	T	37	1	N
12	C-G	24	C	G	36	3	Y
13	A-T	25	A	T	35	2	Y
14	G-C	26	G	C	34	3	Y
15	T-A	27	T	A	33	2	Y
16	G+A	28	G	A	31	2	N

Detailed H-bond formation is as follows:

Pair ID	Pair	No. of pairs	Atom-name pair 1	Distance 1	Atom-name pair 2	Distance 2	Atom-name pair 3	Distance 3
1	G-----C	[3]	N1-N3	3	N2-O2	2.69	O6-N4	3.31
2	C-----G	[3]	O2-N2	3.23	N3-N1	3.03	N4-O6	2.85
3	A-----T	[2]	N1-N3	2.82	N6-O4	3.16		
4	G-----C	[3]	N1-N3	2.98	N2-O2	3.01	O6-N4	3.12
5	C-----G	[3]	O2-N2	2.87	N3-N1	2.87	N4-O6	3.34
6	G-----C	[3]	N1-N3	3.05	N2-O2	2.83	O6-N4	3.14
7	T-----A	[2]	N3-N1	3.13	O4-N6	3.01		
8	T-----A	[2]	N3-N1	2.84	O4-N6	3.58		
9	T-*---T	[2]	N3-O2	3.06	O4-N3	2.85		
10	G-*---T	[1]	N2-O2	2.91				
11	G-*---T	[1]	N2-O2	3.3				

12	C-----G	[3]	O2-N2	2.82	N3-N1	2.86	N4-O6	2.8
13	A-----T	[2]	N1-N3	3.16	N6-O4	3.02		
14	G-----C	[3]	N1-N3	3.02	N2-O2	2.88	O6-N4	2.99
15	T-----A	[2]	N3-N1	2.92	O4-N6	2.75		
16	G-**+-A	[2]	N2-N1	2.75	N3-N6	3.19		

Files generated in this step:

File name	Description
**post_simulation_analysis.xlsx**	A .xlsx file describing local base-pair information

Supplementary note 6: Animal safety data

All animal procedures were performed in the facilities of Science in Action Ltd. (Rehovot, Israel) (National Ethical Approval number 17-3-113). During E8 treatment course signs of stress were monitored including weight **(Supp. Fig. 13)**, physical appearance, alertness, immobility, etc. All animals appeared normal and showed no signs of treatment-related effects.

Supplementary note 7: E8 in human blood

To ensure E8 administration is applicable in a clinical setting, E8 was incubated with blood samples from healthy donors in efficacious doses and several criteria were assessed including blood agglutination **(Supp. Fig. 15)**, red blood cell hemolysis **(Supp. Fig. 16)** and cytokine release from PBMCs via cytokine antibody array kit **(Supp. Fig. 17)**. No effects of agglutination or hemolysis were measured. Only a minor release of certain cytokines was evident, specifically GM-CSF, even at high doses of 100uM. Notably, no adverse runaway immune response was observed in these settings. Representative results are shown.

Supplementary note 8: EVOC experimental design

Ex-vivo organ cultures (EVOC) were prepared by Curesponse **(Supp. Fig. 18)**. vehicle, E8 or standard chemotherapy were administered 1 d after EVOC preparation at 20 or 50 uM. Following 1 d, sample medium was replaced and a second dose at the same concentration was administered. EVOC samples were fixated in 4% w/v paraformaldehyde and histological sections were prepared and stained with hematoxylin and eosin (H&E).

Supplementary note 9: Functional reporter selection

Beads with ssDNA libraries tagged with fluorophore cap, cleaned and counted in a flow cytometer. The beads incubate with target cells at a ratio of about 1:100 (beads : cells, respectively) for one and a half hours and then the cells were washed and taken to stain for apoptotic marker and analyzed with a flow cytometer. Kasumi-1 cells were stained in the SELEX process for Dilc1(5), which detects a change in the mitochondrial membrane potential in cells that can indicate for apoptotic phase. In the MCF-7 cells, the SELEX marker was AnnexinV, a dye that binds to phosphatidylserine that exposes in the outer side of the membrane during the apoptosis process. In HCC1937 cells, calibration was performed before the SELEX process in the presence of beads with an ssDNA library taken from other SELEX and staining with Calcein-AM, which is an indication of active cytoplasmic esterases in living cells.

**Probe Name**	**Distributer**	**CAT#**
CellEvent Caspase-3/7	Invitrogen	C10423
MitoProbe Dilc1(5)	Invitrogen	M34151
Annexin V	BioLegend	640945
VioletRatiometric Membrane Asymmetry	Invitrogen	A35137
Violet Live Cells Caspase	BD Pharmigen	565521
Caspase-8 (active)	abcam	ab65614
Caspase-9 (active	abcam	ab65615
MitoProbe $DiOC_2(3)$	Invitrogen	M34150

CellTrace Calcein Violet	Invitrogen	C34858
