# Supplementary material for: Discovery of tumoricidal DNA oligonucleotides by response-directed in vitro evolution
Source: Commun Biol. 2020 Jan 15;3:29. doi: 10.1038/s42003-020-0756-0 (PMC6962221; doi:10.1038/s42003-020-0756-0)
Supplement: Supplementary file 2 — Reporting Summary [file 42003_2020_756_MOESM2_ESM.pdf]

Corresponding author(s): Ido Bachelet
Last updated by author(s): Nov 8, 2019

nature research

Reporting Summary

Nature Research wishes to improve the reproducibility of the work that we publish. This form provides structure for consistency and transparency in reporting. For further information on Nature Research policies, see Authors & Referees and the Editorial Policy Checklist.

Statistics

For all statistical analyses, confirm that the following items are present in the figure legend, table legend, main text, or Methods section.

n/a	Confirmed	
☐	☒	The exact sample size (*n*) for each experimental group/condition, given as a discrete number and unit of measurement
☐	☒	A statement on whether measurements were taken from distinct samples or whether the same sample was measured repeatedly
☒	☐	The statistical test(s) used AND whether they are one- or two-sided<br>*Only common tests should be described solely by name; describe more complex techniques in the Methods section.*
☒	☐	A description of all covariates tested
☐	☒	A description of any assumptions or corrections, such as tests of normality and adjustment for multiple comparisons
☐	☒	A full description of the statistical parameters including central tendency (e.g. means) or other basic estimates (e.g. regression coefficient) AND variation (e.g. standard deviation) or associated estimates of uncertainty (e.g. confidence intervals)
☒	☐	For null hypothesis testing, the test statistic (e.g. *F*, *t*, *r*) with confidence intervals, effect sizes, degrees of freedom and *P* value noted<br>*Give P values as exact values whenever suitable.*
☒	☐	For Bayesian analysis, information on the choice of priors and Markov chain Monte Carlo settings
☒	☐	For hierarchical and complex designs, identification of the appropriate level for tests and full reporting of outcomes
☒	☐	Estimates of effect sizes (e.g. Cohen's *d*, Pearson's *r*), indicating how they were calculated

Our web collection on statistics for biologists contains articles on many of the points above.

Software and code

Policy information about availability of computer code

Data collection	All custom tools will be made available upon request
Data analysis	All custom tools will be made available upon request

For manuscripts utilizing custom algorithms or software that are central to the research but not yet described in published literature, software must be made available to editors/reviewers. We strongly encourage code deposition in a community repository (e.g. GitHub). See the Nature Research guidelines for submitting code & software for further information.

Data

Policy information about availability of data

All manuscripts must include a data availability statement. This statement should provide the following information, where applicable:

- Accession codes, unique identifiers, or web links for publicly available datasets
- A list of figures that have associated raw data
- A description of any restrictions on data availability

All raw data and data analysis tools will be made available upon request

Field-specific reporting

Please select the one below that is the best fit for your research. If you are not sure, read the appropriate sections before making your selection.

☒ Life sciences ☐ Behavioural & social sciences ☐ Ecological, evolutionary & environmental sciences

For a reference copy of the document with all sections, see nature.com/documents/nr-reporting-summary-flat.pdf

Life sciences study design

All studies must disclose on these points even when the disclosure is negative.

Sample size	Samples were simply determined as number of separate samples on which process was run; for flow cytometry number of events collected per experiment was used as sample size
Data exclusions	No data was excluded
Replication	We specifically describe number of reproducible runs out of total runs in this study
Randomization	N/A
Blinding	Testing of effect on human tumors was analyzed by two blinded pathologists (did not know which treatment was given to which sample)

Reporting for specific materials, systems and methods

We require information from authors about some types of materials, experimental systems and methods used in many studies. Here, indicate whether each material, system or method listed is relevant to your study. If you are not sure if a list item applies to your research, read the appropriate section before selecting a response.

Materials & experimental systems

n/a	Involved in the study	
☐	☒	Antibodies
☐	☒	Eukaryotic cell lines
☒	☐	Palaeontology
☐	☒	Animals and other organisms
☐	☒	Human research participants
☒	☐	Clinical data

Methods

n/a	Involved in the study	
☒	☐	ChIP-seq
☐	☒	Flow cytometry
☒	☐	MRI-based neuroimaging

Antibodies

Antibodies used	Commercial antibodies were used as described in the manuscript
Validation	Antibodies were used according to the manufacturer's instructions and validated by FACS

Eukaryotic cell lines

Policy information about cell lines

Cell line source(s)	Cell lines were either acquired from American Type Cell Culture, or given by a collaborating doctor as described
Authentication	Cell lines were not authenticated
Mycoplasma contamination	Samples were regularly probed for Mycoplasma contamination, found negative
Commonly misidentified lines (See ICLAC register)	N/A

Animals and other organisms

Policy information about studies involving animals; ARRIVE guidelines recommended for reporting animal research

Laboratory animals	C57BL6 mice
Wild animals	N/A
Field-collected samples	N/A
Ethics oversight	All animal experiments were reviewed and approved by the national committee for ethics in animal experimentation

Note that full information on the approval of the study protocol must also be provided in the manuscript.

Human research participants

Policy information about studies involving human research participants

Population characteristics	N/A
Recruitment	Human primary acute myeloblastic leukemia (AML) cells were isolated from patients by standard procedures (Institutional Review Board [IRB] approval numbers 0297-15-TLV & 4573-17-SMC). Human primary PBMCs were isolated from healthy donors by standard procedures (IRB approval number 0297-15-TLV). All participants signed informed consent.
Ethics oversight	See above

Note that full information on the approval of the study protocol must also be provided in the manuscript.

Flow Cytometry

Plots

Confirm that:

☒ The axis labels state the marker and fluorochrome used (e.g. CD4-FITC).

☒ The axis scales are clearly visible. Include numbers along axes only for bottom left plot of group (a 'group' is an analysis of identical markers).

☒ All plots are contour plots with outliers or pseudocolor plots.

☒ A numerical value for number of cells or percentage (with statistics) is provided.

Methodology

Sample preparation	Target blasts were isolated by magnetic sorting using a commercial kit (Miltenyi Biotec) according to the manufacturer's instructions.
Instrument	Sorting was performed on a Becton-Dickinson FACSMelody cell sorter equipped with blue, red, and violet lasers (9 color configuration). Flow cytometry was performed on a Becton-Dickinson Accuri C6 Plus cytometer equipped with 488 nm and 630 nm lasers, and on a Beckman-Coulter Cytoflex cytometer with a B5-R3-V5 laser configuration.
Software	We used Kaluza throughout the study
Cell population abundance	Depending on sample
Gating strategy	N/A

☒ Tick this box to confirm that a figure exemplifying the gating strategy is provided in the Supplementary Information.
